# Statin Use in Patients With Advanced Prostate Cancer in the TITAN and SPARTAN Trials

**DOI:** 10.1001/jamanetworkopen.2025.27988

**Published:** 2025-08-20

**Authors:** Soumyajit Roy, Zeynep Irem Ozay, Avirup Guha, Yilun Sun, Kim N. Chi, Scott C. Morgan, Shawn Malone, Christopher J. D. Wallis, Jason R. Brown, Pedro C. Barata, Amar U. Kishan, Angela Y. Jia, Nicholas G. Zaorsky, Umang Swami, Simon Chowdhury, Eric J. Small, Daniel E. Spratt, Fred Saad, Neeraj Agarwal

**Affiliations:** 1 Department of Radiation Oncology, University Hospitals Seidman Cancer Center, Case Western Reserve University, Cleveland, Ohio; 2 Department of Medical Oncology, Huntsman Cancer Institute, University of Utah, Salt Lake City; 3 Cardio-Oncology Program, Georgia Cancer Center, Augusta University, Augusta, Georgia; 4 Division of Cardiology, Department of Medicine, Medical College of Georgia, Augusta; 5 Department of Medical Oncology, BC Cancer – Vancouver, University of British Columbia, Vancouver, British Columbia, Canada; 6 Department of Radiation Oncology, Medical Physics, and Radiology, The Ottawa Hospital Cancer Centre, University of Ottawa, Ottawa, Ontario, Canada; 7 Division of Urology, Mount Sinai Hospital, University Hospital Network, University of Toronto, Toronto, Ontario, Canada; 8 Department of Internal Medicine Division of Solid Tumor Oncology, University Hospitals Seidman Cancer Center, Case Western Reserve University, Cleveland, Ohio; 9 Department of Radiation Oncology, University of California, Los Angeles; 10 Guy’s and St Thomas’ NHS Foundation Trust and Sarah Cannon Research Institute, London, United Kingdom; 11 Department of Medicine, University of California, San Francisco; 12 Department of Surgery, Centre Hospitalier de l’Université de Montréal, Montréal, Quebec, Canada

## Abstract

**Importance:**

The impact of statins on overall survival (OS) in patients with prostate cancer treated using intensified therapy with apalutamide is not fully understood.

**Objective:**

To determine whether statin exposure was associated with OS and grade 3 or greater cardiac adverse events (AEs) in patients treated with apalutamide.

**Design, Setting, and Participants:**

This cohort study used individual patient data from 2 multicenter phase 3 randomized clinical trials, SPARTAN (October 14, 2013, to December 15, 2016) and TITAN (December 15, 2015, to July 25, 2017). The final analysis was done on May 21, 2025. TITAN and SPARTAN randomized patients to receive androgen deprivation therapy with or without apalutamide in metastatic hormone-sensitive prostate cancer (TITAN) and nonmetastatic castration-resistant prostate cancer (SPARTAN).

**Exposure:**

Statin exposure during the assigned treatment; statin exposure could have started before and ended either during or after the assigned treatment.

**Main Outcomes and Measures:**

Inverse probability of treatment–weighted (IPTW) multivariable Cox proportional hazard regression model and covariate-adjusted OS were estimated for patients with and without statin exposure in each trial. Fine and Gray regression models were applied to determine the association of statin exposure with risk of grade 3 or greater cardiac AEs, considering deaths as competing events.

**Results:**

Among 2187 patients included in this analysis, 1288 received apalutamide (517 in TITAN; 770 in SPARTAN) and 900 received placebo. The median (IQR) age was 65 (60-70) years in TITAN and 70 (65-80) years in SPARTAN. Patients with statin exposure were older with higher body mass index, while the proportion of patients with ECOG performance status score 1 was relatively lower. Statin exposure was associated with superior OS in patients treated with apalutamide (TITAN: hazard ratio [HR], 0.53; 95% CI, 0.32-0.87; SPARTAN: HR, 0.54; 95% CI, 0.39-0.74), but not in patients who received the placebo (TITAN: HR, 0.65; 95% CI, 0.38-1.13; SPARTAN: HR, 1.16; 95% CI, 0.76-1.77). Covariate-adjusted 3-year OS among patients with vs without statins were 81% vs 67% (difference, 14% [95% CI, 5%-22%]) and 86% vs 78% (difference, 8% [95% CI, 3%-13%]) in the apalutamide-treated patients from TITAN and SPARTAN trials, respectively. Patients receiving statins had a higher risk of grade 3 or higher cardiac AEs in both apalutamide (subdistribution HR, 2.62 [95% CI, 1.35-5.08]) and placebo (subdistribution HR, 2.36 [95% CI, 0.96-5.84]) groups.

**Conclusions and Relevance:**

In this cohort study, statin exposure was associated with longer OS in patients treated with apalutamide. Statin-exposed patients had a higher risk of grade 3 or greater cardiac AEs, which may reflect their preexisting cardiovascular comorbidity.

## Key Points

**Question:**

Is statin exposure associated with overall survival and cardiac adverse events in men with advanced prostate cancer treated with apalutamide?

**Findings:**

This cohort study using individual patient data from 2187 patients in the SPARTAN and TITAN randomized clinical trials found that statin exposure was associated with superior overall survival among all apalutamide-treated patients, although patients receiving statins had a higher risk of grade 3 or greater cardiac adverse events, possibly reflecting their preexisting cardiovascular comorbidity.

**Meaning:**

These findings suggest a possible role of statin exposure in patients with advanced prostate cancer treated with androgen blockade.

## Teaser

This cohort study uses individual patient data from 2 randomized clinical trials of advanced prostate cancer to examine the associations of statin exposure with overall survival and adverse events in patients treated with apalutamide.

## Introduction

Statins are competitive inhibitors of cholesterol biosynthesis and additional derivatives of the mevalonate pathway. They work by inhibiting the enzyme 3-hydroxy-3-methylglutaryl-coenzyme A reductase, which is the rate-limiting step in cholesterol biosynthesis in the liver.^1^ By virtue of this mechanism of action, they are pivotal to cardiovascular risk prevention strategies.^2,3^ Beyond this major health benefit, an evolving body of evidence also suggests a possible role of statins in prostate cancer prevention or forestalling prostate cancer progression.^4^ Statin use was associated with lower prostate cancer–specific mortality among men undergoing androgen deprivation therapy (ADT) in the Finnish Randomized Study for Prostate Cancer Screening.^5^ Similar findings have been observed in studies that included patients with advanced prostate cancer treated with androgen-receptor pathway inhibitors (ARPI).^6,7,8^ A pooled analysis of the PROSPER, AFFIRM, and PREVAIL trials and a separate pooled analysis of the AFFIRM and PREVAIL trials in later phases of advanced prostate cancer, ie, metastatic castration-resistant prostate cancer (mCRPC), found that concurrent statin use was associated with improved overall survival (OS) in patients treated with enzalutamide.^6^ Similarly, in another pooled analysis of the COU-AA-301 and AA-302 trials, OS was longer among statin-exposed patients with mCRPC treated with abiraterone.^8^ While grouped under the umbrella of ARPI agents, agents such as enzalutamide, darolutamide, or apalutamide have varying drug-drug interactions and safety profiles, in addition to variable efficacy, as noted in clinical studies.^9,10,11^ The effect of statins on patients treated with apalutamide, especially in earlier stages of advanced prostate cancer, such as metastatic hormone-sensitive prostate cancer (mHSPC) or nonmetastatic castration-resistant prostate cancer (nmCRPC) remains to be elucidated. In this study, we performed an individual patient data–based analysis of the SPARTAN and TITAN trials to determine the associations of statin exposure with OS and grade 3 or greater cardiac adverse events (AEs) in patients with advanced prostate cancer treated with apalutamide.

## Methods

For this cohort study, ethical review and informed consent were waived by the institutional review committee of the Ottawa Hospital per the data sharing agreement with the data sharing platforms. This study is reported following the Strengthening the Reporting of Observational Studies in Epidemiology (STROBE) reporting guideline.

This is a pooled analysis of individual patient data from 2 phase 3 randomized clinical trials that established the role of apalutamide in patients with nmCRPC (SPARTAN) and those with mHSPC (TITAN). The TITAN trial (NCT02489318) was a multicentric phase 3 double-blinded, placebo-controlled randomized clinical trial in which men with mHSPC were randomly assigned to receive apalutamide or matched placebo in conjunction with ADT at a 1:1 ratio.^12,13^ Compared with placebo, apalutamide plus ADT significantly reduced the risk of death by 35% (median OS, 52.2 months vs not reached; hazard ratio [HR], 0.65; 95% CI, 0.53-0.79) and by 48% after adjustment for crossover (HR, 0.52; 95% CI, 0.42-0.64).^12^

The SPARTAN trial (NCT01946204) was a phase 3 multicenter randomized clinical trial conducted between October 14, 2013, and December 15, 2016.^14,15^ Eligible patients were aged at least 18 years with nmCRPC and prostate-specific antigen (PSA) doubling time of at least 10 months during continuous ADT. Patients were randomly assigned in a 2:1 ratio to ADT plus apalutamide or ADT plus placebo. The addition of apalutamide to ADT led to a significant improvement in metastasis-free survival (HR, 0.28; 95% CI, 0.23-0.35).^14^ At a median follow-up of 52 months, OS was significantly longer with ADT plus apalutamide compared with ADT alone (HR, 0.78; 95% CI, 0.64-0.96).^15^

For this study, patients were considered exposed to statins if they had evidence of use at any time during their assigned treatment period. This included patients who initiated statins before or during the study-assigned treatment and continued their use during or beyond the assigned treatment period.

### Statistical Analysis

Descriptive statistics were used to characterize the study cohort. To determine differential associations of statin exposure with OS in the apalutamide and placebo groups, we applied a mixed-effects Cox proportional hazard regression model with an interaction between statin exposure and treatment as a fixed-effect covariate and a random slope across the 2 studies along with stratification for the study (TITAN vs SPARTAN). Additional fixed-effect covariates included age; body mass index (BMI); Eastern Cooperative Oncology Group (ECOG) performance status; Gleason score (≤7 vs 8-10 vs unknown); presence of diabetes, dyslipidemia, vascular disorders (ie, presence of arteriosclerosis, ischemic heart disease, coronary artery disease, peripheral arterial disorders, or cerebrovascular disorders), or hypertension; tumor stage (T1-T2 vs T3-T4 vs unknown), and race (Asian, Black, White, or others). In the 2 trials, race was originally categorized as American Indian or Alaska Native, Asian, Black, White, unreported, and others. For this analysis, American Indian or Alaska Native, unreported, and others were grouped together due to very small sample sizes. Subsequently, we determined the association of statin exposure with OS across the 2 treatment groups (apalutamide and placebo groups) accounting for statin exposure as a fixed-effect covariate and as a random slope across the studies. Proportionality assumption for the Cox models was tested by visually inspecting the Schoenfeld residuals.

Subsequently standard multivariable Cox proportional hazard regression models with and without inverse probability of treatment weighting (IPTW) were applied to determine the association of statin exposure with hazards of deaths in the 2 treatment groups of the TITAN and SPARTAN trials separately. Furthermore, covariate-adjusted 3-year OS based on direct standardization (also known as the *G-computation* or *corrected group prognosis method*) was compared among patients with and without statin exposure within each of the 2 treatment groups of the SPARTAN and TITAN trials. These covariate-adjusted survival probabilities were estimated by standardizing across the distribution of observed covariates, which is a well-established approach for estimating marginal survival curves.^16,17^ Given the prognostic implication of visceral metastasis, number of skeletal lesions, and metastatic stage at presentation (synchronous vs asynchronous); baseline PSA; disease volume (CHAARTED definition^18^); and risk group (LATITUDE definition^19^) in mHSPC, we included these as additional confounders for analyses limited to the TITAN trial. As a sensitivity analysis, we conducted landmark sensitivity analyses at 6, 12, and 24 months. We restricted the cohort to patients who were alive and uncensored at each time point and defined statin exposure based on whether patients had ongoing or completed statin therapy by that time. OS was then measured from the landmark onward, and the association of statin exposure with OS was measured using standard multivariable Cox proportional hazard models adjusted for baseline covariates. Time-to-event end points were estimated from time of random assignment.

To characterize the association of statin exposure with risk of grade 3 or greater cardiac AEs, we applied Fine and Gray multivariable regression models in which deaths were considered competing events and patients without events were censored at the date of last follow-up. Age, BMI, history of diabetes, vascular disorders, hypertension, and trial (SPARTAN vs TITAN) were considered as fixed covariates in addition to an interaction term between treatment and statin exposure. Subsequently, the association of statin exposure with risk of grade 3 or greater cardiac AEs was determined across treatment regimens within the 2 trials separately. Furthermore, we applied multivariable logistic regression models to determine the association of statin exposure with overall odds of grade 3 or greater cardiac AEs. No formal adjustment of *P* values was performed to account for multiple testing, and 2-sided *P* < .05 was considered significant. All analyses were conducted using R software version 4.4.1 (R Project for Statistical Computing) with its statistical packages. The final analysis was done on May 21, 2025.

## Results

Overall, 2187 patients were included in this study, of whom 1039 were from the TITAN trial (median [IQR] age, 65 [60-70] years) and 1148 were from the SPARTAN study (median [IQR] age, 70 [65-80] years); 1288 received apalutamide (517 in TITAN; 770 in SPARTAN) and 900 received placebo (eFigure in Supplement 1). Overall, 748 patients (34.2%) were exposed to statins. When stratified by study, 242 patients (23.3%) in the TITAN trial and 506 patients (44.1%) in the SPARTAN trial were exposed to statins. Patients with statin exposure were older and had higher BMI, while the proportion of patients with ECOG performance status score of 1 was lower compared with unexposed patients. More patients had history of diabetes (105 patients [14.1%] vs 115 patients [8.0%]), dyslipidemia (428 patients [57.1%] vs 133 patients [9.6%]), hypertension (570 patients [76.1%] vs 774 patients [53.8%]), and vascular disorders (165 patients [22.0%] vs 110 patients [7.6%]) in the statin-exposed group compared with the unexposed group. Baseline characteristics are summarized in Table 1.

**Table 1.** Baseline Characteristics

Characteristic	Participants, No. (%)				*P* value
	Statin exposure		No statin exposure		
	TITAN (n = 242)	SPARTAN (n = 506)	SPARTAN (n = 642)	TITAN (n = 797)	
Age, median (IQR), y	70.0 (65.0-75.0)	70.0 (65.0-80.0)	70.0 (65.0-75.0)	65.0 (60.0-70.0)	<.001
BMI, median (IQR)	26.8 (24.5-30.0)	29.1 (26.2-32.6)	27.7 (24.8-31.1)	25.8 (23.2-28.4)	<.001
Race					
Asian	49 (20.2)	39 (7.7)	99 (15.4)	181 (22.7)	<.001
Black	6 (2.5)	39 (7.7)	24 (3.7)	13 (1.6)	
Others	15 (6.2)	71 (14.0)	108 (16.8)	68 (8.5)	
White	172 (71.1)	357 (70.6)	411 (64.1)	535 (67.1)	
ECOG performance status					
0	173 (71.5)	379 (74.9)	491 (76.5)	495 (62.1)	<.001
1	69 (28.5)	127 (25.1)	151 (23.5)	302 (37.8)	
Treatment					
Apalutamide	139 (57.4)	344 (68.0)	426 (66.1)	378 (47.4)	<.001
Placebo	103 (42.6)	162 (32.0)	216 (33.9)	419 (52.6)	
Nodal stage					
N0	115 (47.5)	356 (70.4)	434 (67.7)	310 (38.9)	<.001
N1	73 (30.2)	66 (13.0)	107 (16.6)	304 (38.1)	
NX	54 (22.3)	84 (16.6)	101 (15.7)	183 (23.0)	
Tumor stage					
T1-T2	82 (33.9)	270 (53.4)	304 (47.4)	241 (30.2)	<.001
T3-T4	136 (56.2)	236 (46.6)	338 (52.6)	472 (59.2)	
TX	24 (9.9)	0	0	84 (10.5)	
Gleason score					
≤7	81 (33.5)	292 (57.7)	337 (52.5)	259 (32.5)	<.001
>7	161 (66.5)	203 (40.1)	289 (44.7)	538 (67.5)	
Unknown	0	11 (2.2)	18 (2.8)	0	
Diabetes					
No	211 (87.2)	432 (85.4)	584 (90.8)	741 (93.0)	<.001
Yes	31 (12.8)	74 (14.6)	58 (9.2)	56 (7.0)	
Dyslipidemia					
No	98 (40.5)	222 (44.0)	544 (84.7)	762 (95.6)	<.001
Yes	144 (59.5)	284 (56.0)	98 (15.3)	35 (4.4)	
Vascular disorders					
No	192 (79.3)	391 (77.3)	597 (93.0)	732 (91.8)	<.001
Yes	50 (20.7)	115 (22.7)	45 (7.0)	65 (8.2)	
Hypertension					
No	70 (28.8)	108 (21.3)	250 (38.9)	415 (52.1)	<.001
Yes	172 (71.2)	398 (78.7)	392 (61.1)	382 (47.9)	

Abbreviations: BMI, body mass index (calculated as weight in kilograms divided by height in meters squared); ECOG, Eastern Cooperative Oncology Group.

Median (IQR) follow-up times for patients with and without statin exposure were 49 (44-57) months and 46 (41-52) months, respectively. In the TITAN trial, median (IQR) follow-up times for patients with and without statin exposure were 44 (41-47) months and 44 (41-46) months, respectively. In the TITAN trial, 403 deaths were recorded: 35 of 103 patients in the statin-exposed subgroup in the placebo group, 32 of 139 patients in the statin-exposed subgroup in the apalutamide group, 199 of 419 patients in the non–statin exposed subgroup in the placebo group, and 137 of 378 patients in the non–statin exposed subgroup in the apalutamide group. In the SPARTAN trial, median (IQR) follow-up times for patients with and without statin exposure were 51 (44-59) months and 53 (47-59) months, respectively. In the SPARTAN trial, 405 deaths were recorded: 64 of 162 patients in the statin-exposed subgroup in the placebo group, 104 of 345 patients in the statin-exposed subgroup in the apalutamide group, 79 of 218 patients in the non–statin exposed subgroup in the placebo group, and 158 of 426 patients in the non–statin exposed subgroup in the apalutamide group. Causes of deaths are summarized by statin exposure in Table 2.

**Table 2.** Causes of Deaths Across Studies by Statin Exposure

Statin Exposure	Participants, No. (%)			
	Alive	Dead		
		Adverse events	Other causes	Progressive disease
**SPARTAN**				
Without Statins				
Apalutamide	268 (63.0)	12 (2.8)	47 (11.0)	99 (23.3)
Placebo	137 (63.4)	1 (0.5)	22 (10.2)	56 (25.9)
With Statins				
Apalutamide	240 (69.8)	8 (2.3)	33 (9.6)	63 (18.3)
Placebo	98 (60.5)	5 (3.1)	20 (12.3)	39 (24.1)
**TITAN**				
Without Statins				
Apalutamide	241 (63.8)	19 (5.0)	22 (5.8)	96 (25.4)
Placebo	220 (53.1)	23 (5.5)	34 (8.2)	142 (34.3)
With Statins				
Apalutamide	107 (77.0)	5 (3.6)	9 (6.5)	18 (12.9)
Placebo	68 (66.0)	3 (2.9)	8 (7.8)	24 (23.3)

In the mixed-effects Cox model, we did not find any statistically significant heterogeneity in the association of statin use with OS across treatment groups (*P* for interaction = .47), with a very minimal study-level heterogeneity in the interaction (variance, 0.0002). In the overall study cohort, statin exposure was associated with significantly improved OS (HR, 0.67; 95% CI, 0.48-0.86). In patients who were treated with apalutamide, statin exposure was associated with a 42% reduction in the hazard of death (HR, 0.58; 95% CI, 0.42-0.92), while in the placebo group, statin exposure was associated with a 17% reduction in the hazard of death (HR, 0.83; 95% CI, 0.32-1.02). The study-level random variance was very low, at 0.00006 and 0.020 for the apalutamide and placebo groups, respectively, suggesting lack of substantial interstudy heterogeneity.

Subsequently, we explored the association of statin exposure with OS in the apalutamide and placebo groups of the TITAN and SPARTAN trials. Statin exposure was associated with significant improvement in OS in patients treated with apalutamide in both TITAN and SPARTAN trials on multivariable Cox models with and without IPTW (Table 3). In contrast, on IPTW-based analysis in the placebo group in the TITAN trial, statin exposure was not associated with OS in patients with mHSPC (HR, 0.65; 95% CI, 0.38 to 1.13). Similarly, there was no significant association of statin exposure with OS in patients with nmCRPC in the placebo group of the SPARTAN trial (HR, 1.16; 95% CI, 0.77 to 1.77) (Table 3). In the patients treated with apalutamide from the TITAN trial, 3-year covariate-adjusted OS rates for patients with vs without statin exposure were 81% (95% CI, 74% to 88%) vs 67% (95% CI, 62% to 72%), with a significant difference between groups (adjusted difference, 14%; 95% CI, 5% to 22%) (Figure, A). In the patients treated with apalutamide from SPARTAN trial, 3-year covariate-adjusted OS rates for patients with vs without statin exposure were 86% (95% CI, 83% to 89%) vs 78% (95% CI, 74% to 82%), with a significant difference between groups (adjusted difference, 8%; 95% CI, 3% to 13%) (Figure, B). In the patients treated with placebo from the TITAN trial, 3-year covariate-adjusted OS rates for patients with vs without statin exposure were 70% (95% CI, 61% to 80%) vs 58% (95% CI, 53% to 63%), with a significant difference between groups (adjusted difference, 12%; 95% CI, 2% to 23%) (Figure, C), while in the patients treated with placebo from SPARTAN trial, 3-year covariate-adjusted OS rates for patients with vs without statin exposure were 75% (95% CI, 68% to 81%) vs 77% (95% CI, 72%-83%), with no significant difference between groups (adjusted difference, −3%; 95% CI, −11% to 6%) (Figure, D). On multivariable Cox proportional hazard regression model in landmark populations, the findings were consistent with the primary analysis, with longer OS among statin-exposed subgroups treated with apalutamide in both TITAN and SPARTAN trials (eTable 1 in Supplement 1).

**Table 3.** Trial-Specific Associations of Statin Exposure With OS Across Treatment Regimens

Treatment group	Estimate (95% CI)	
	TITAN	SPARTAN
**OS HR**		
Apalutamide		
Without IPTW	0.51 (0.31-0.84)	0.59 (0.44-0.80)
With IPTW	0.53 (0.32-0.87)	0.54 (0.39-0.74)
Placebo		
Without IPTW	0.60 (0.37-0.97)	1.14 (0.76-1.72)
With IPTW	0.65 (0.38-1.13)	1.16 (0.77-1.77)
**Covariate-adjusted 3-y OS, %** ^a^		
Apalutamide		
With statins	81 (74-88)	86 (83-89)
Without statins	67 (62-72)	78 (74-82)
Placebo		
With statins	70 (61-80)	75 (68-81)
Without statins	58 (53-63)	78 (72-83)

Abbreviations: HR, hazard ratio; IPTW, Inverse probability of treatment weighting; OS, overall survival.

^a^ Confounders that were common to both trials were age; body mass index; Eastern Cooperative Oncology Group performance status; Gleason score (≤7 vs 8-10 vs unknown); presence of diabetes, dyslipidemia, vascular disorders (ie, presence of arteriosclerosis, ischemic heart disease, coronary artery disease, peripheral arterial disorders, or cerebrovascular disorders), and hypertension; tumor stage (T1-T2 vs T3-T4 vs unknown), and race (Asian, Black, White, and others). Additional confounders that were unique to the TITAN trial were visceral metastasis, number of skeletal lesions, and metastatic stage at presentation (synchronous vs asynchronous), baseline PSA, disease volume (CHAARTED definition), and risk group (LATITUDE definition).

**Figure.**
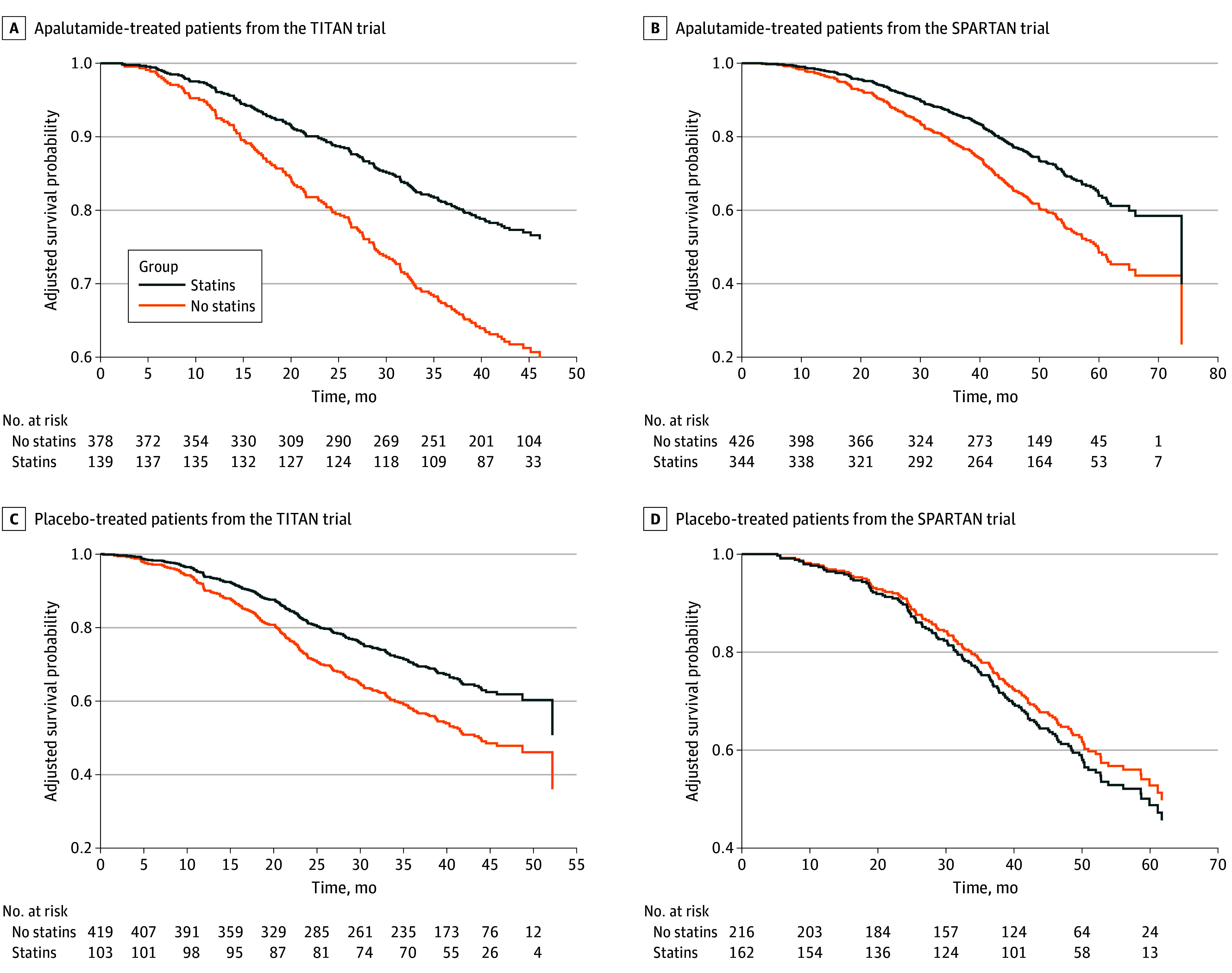
Confounder-Adjusted Overall Survival Curves for Patients With and Without Statin Exposure in Apalutamide and Placebo Groups From the TITAN and SPARTAN Trials

Overall distribution of grade 3 or greater AEs across the 2 trials have been summarized in eTable 2 and eTable 3 in Supplement 1. Among patients with statin exposure, grade 3 or greater cardiac AEs were noted in 28 patients (5.8%) in the apalutamide cohort and 12 patients (4.5%) within the placebo cohort. Among patients without statin exposure, 17 (2.1%) in the apalutamide cohort and 8 (1.2%) in the placebo cohort had grade 3 or greater cardiac AEs. Statin exposure was associated with higher relative incidence of grade 3 or greater cardiac AEs in both apalutamide and placebo groups, with no significant difference between treatment groups (*P* for interaction = .65). Patients with statin exposure were found to have increased risk of grade 3 or greater cardiac AEs in both apalutamide (subdistribution HR, 2.57; 95% CI, 1.32-5.00) and placebo (subdistribution HR, 2.34; 95% CI, 0.97-5.78) groups. The findings were consistent when stratified by trials (Table 4). The findings from the logistic regression model are summarized in eTable 4 in Supplement 1.

**Table 4.** Overall and Trial-Specific Association of Statin Exposure With Risk of Grade 3 or Greater Cardiac Adverse Events Across Treatment Regimens

Treatment group	Subdistribution hazard ratio (95% CI)		
	Overall	TITAN	SPARTAN
Apalutamide	2.57 (1.32-5.00)	2.68 (0.97-7.37)	2.55 (1.05-6.22)
Placebo	2.34 (0.95-5.78)	1.94 (0.55-6.84)	2.81 (0.75-10.60)

## Discussion

In this retrospective post hoc cohort study of patient-level data from 2 phase 3 randomized clinical trials, statin exposure was associated with longer OS in men with advanced prostate cancer treated with apalutamide. Patients receiving statins had a higher risk of grade 3 or greater cardiac AEs in the apalutamide group, which could reflect a spurious association or reverse causality due to residual confounding from higher preexisting burden of cardiac or noncardiac comorbidity, which was not captured with sufficient granularity in the data. Furthermore, use of statins in these patients might reflect a measure to mitigate cardiac AEs (ie, an indication bias) and minimize non–cancer related deaths among patients treated with intensified hormonal therapy using ARPIs (like apalutamide).^20,21^ The crude proportional estimates of causes of deaths support this hypothesis. In our study, statin exposure was associated with a lower rate of deaths from cancer progression, especially in the apalutamide group, while patients receiving statins had a similar crude rate of non–cancer related deaths even after being treated with more profound androgen blockade. This could explain a relatively higher absolute covariate-adjusted OS benefit associated with statin exposure among patients treated with apalutamide in our study.

While our findings are hypothesis generating, there are some plausible biologic mechanisms that could explain the underlying interaction of apalutamide with statins that we observed in these patients with relatively earlier stages of advanced prostate cancer compared with the mCRPC setting. Statins could reduce risk of prostate cancer progression and cancer-specific mortality in patients treated with ARPI, which is mediated through lipid-lowering and non–lipid lowering mechanisms.^4,22,23,24^ The cholesterol-mediated mechanisms are postulated to involve cell signaling processes related to androgen receptors (ARs), and epidermal growth factor receptors while non–cholesterol mediated mechanisms are related to the downregulating effect of statins on cell proliferation and adhesion, angiogenesis, and inflammation and selective upregulation of apoptosis in tumor cells.^23,24^ Cholesterol-rich membrane rafts are potential sites of nongenomic signal processing and transduction, which could drive disease progression in prostate cancers that are AR driven; however, these might not be evident in prostate cancers that are AR independent.^25^ The possible effect of statins on these lipid-rich membrane rafts could explain our finding of longer OS among patients with statin exposure in the ADT-treated mHSPC group, for whom cancer is strongly driven by androgen signaling, compared with no significant OS benefit in patients with nmCRPC in the placebo group. Another important aspect that was unexplored in our study is a possible interaction in mHSPC between metformin and statins, which are often simultaneously used in patients who have metabolic syndromes.^26^ Metformin was not associated with reduction in the hazard of mortality in patients with mHSPC in the STAMPEDE trial.^27^ Future studies should examine how the potential interaction between these 2 commonly prescribed medications affect outcomes in mHSPC.

### Limitations

This study has some limitations, and some of our observed findings could reflect underlying bias and unmeasured confounding. Some of the limitations of our study include inadequate power to detect between-group differences, higher risk of false discovery rates despite adjusting for multiple analyses, lack of information on serial serum cholesterol levels, residual unmeasured confounding, and residual selection bias, among others. For the purposes of this analysis, we focused on exposure rather than cumulative dose or duration, especially due to lack of information on start and end dates for statin exposure. Therefore, a dose-dependent association could not be established. Furthermore, the lack of information on specific statin agents raises concerns for misclassification bias. Additionally, we could not investigate potential drug-drug interactions, which is often noted between apalutamide and statins^28,29^ and could potentially explain the increased risk of cardiac AEs in our study cohort. Our OS findings could be liable to immortal time bias. Given that concomitant exposure was defined as receipt of the concomitant medications at baseline or at any point while receiving the randomly assigned treatment regimen, the time until the patient was not exposed to a particular medication in the exposed group could result in false inflation of time to event. A number of these patients were probably exposed to more than 1 class of concomitant medication, which was not explored in our analysis. Furthermore, statins could be used as primary prevention by some patients, especially in this age group. Therefore, statin exposure may reflect health-seeking behaviors that may have independent survival benefits, including prostate cancer–specific outcomes, as these behaviors could also indicate better adherence to prostate cancer treatment leading to improved cancer-specific outcomes. Additionally, representation from some of racial and ethnic minority groups (eg, American Indian, Alaska Native, or Black) was extremely low in our study population, and this limits the generalizability of our findings. Overall, our findings should be interpreted with caution.

## Conclusions

In this cohort study including patients from 2 randomized clinical trials, statin exposure during the randomly assigned treatment regimen was associated with a longer overall survival in patients with advanced prostate cancer treated with more profound androgen blockade with apalutamide. Patients receiving statins had a higher risk of grade 3 or greater cardiac adverse events, which may be a reflection of their preexisting cardiovascular comorbidity. Given the post hoc exploratory nature of the study, these findings are at best hypothesis generating, and need further validation in additional studies.

## Notes

Supplement 1. **eFigure.** Participant recruitment flowchart **eTable 1.** Multivariable Cox proportional hazard regression model summarizing association of statin exposure with OS among patients treated with and without apalutamide in the TITAN and SPARTAN trials in landmark populations at various timepoints **eTable 2.** Distribution of organ systemic specific grade ≥3 adverse events in apalutamide treated patients stratified by exposure to statins **eTable 3.** Distribution of organ systemic specific grade ≥3 adverse events in placebo treated patients stratified by exposure to statins **eTable 4.** Overall and trial specific association of statin exposure with risk of grade ≥3 cardiac adverse events (SAE) across 2 different treatment regimens using logistic regression model

Supplement 2.
Data Sharing Statement
