# Supplementary material for: Statin Use in Patients With Advanced Prostate Cancer in the TITAN and SPARTAN Trials
Source: JAMA Netw Open. 2025 Aug 20;8(8):e2527988. doi: 10.1001/jamanetworkopen.2025.27988 (PMC12368675; doi:10.1001/jamanetworkopen.2025.27988)
Supplement: Supplement 1. — eFigure. Participant recruitment flowchart eTable 1. Multivariable Cox proportional hazard regression model summarizing association of statin exposure with OS among patients treated with and without apalutamide in the TITAN and SPARTAN trials in landmark populations at various timepoints eTable 2. Distribution of organ systemic specific grade ≥3 adverse events in apalutamide treated patients stratified by exposure to statins eTable 3. Distribution of organ systemic specific grade ≥3 adverse events in placebo treated patients stratified by exposure to statins eTable 4. Overall and trial specific association of statin exposure with risk of grade ≥3 cardiac adverse events (SAE) across 2 different treatment regimens using logistic regression model [file jamanetwopen-e2527988-s001.pdf]

Supplemental Online Content

Roy S, Ozay ZI, Guha A, et al. Statin use in patients with advanced prostate cancer in the TITAN and SPARTAN trials. *JAMA Netw Open*. 2025;8(8):e2527988. doi:10.1001/jamanetworkopen.2025.27988

eFigure. Participant Recruitment Flowchart

eTable 1. Multivariable Cox proportional hazard regression model summarizing association of statin exposure with OS among patients treated with and without apalutamide in the TITAN and SPARTAN trials in landmark populations at various timepoints

eTable 2. Distribution of organ systemic specific grade ≥3 adverse events in apalutamide treated patients stratified by exposure to statins

eTable 3. Distribution of organ systemic specific grade ≥3 adverse events in placebo treated patients stratified by exposure to statins

eTable 4. Overall and trial specific association of statin exposure with risk of grade ≥3 cardiac adverse events (SAE) across 2 different treatment regimens using logistic regression model

This supplemental material has been provided by the authors to give readers additional information about their work.

© 2025 Roy S et al. *JAMA Network Open*.

eFigure 1: CONSORT Diagram

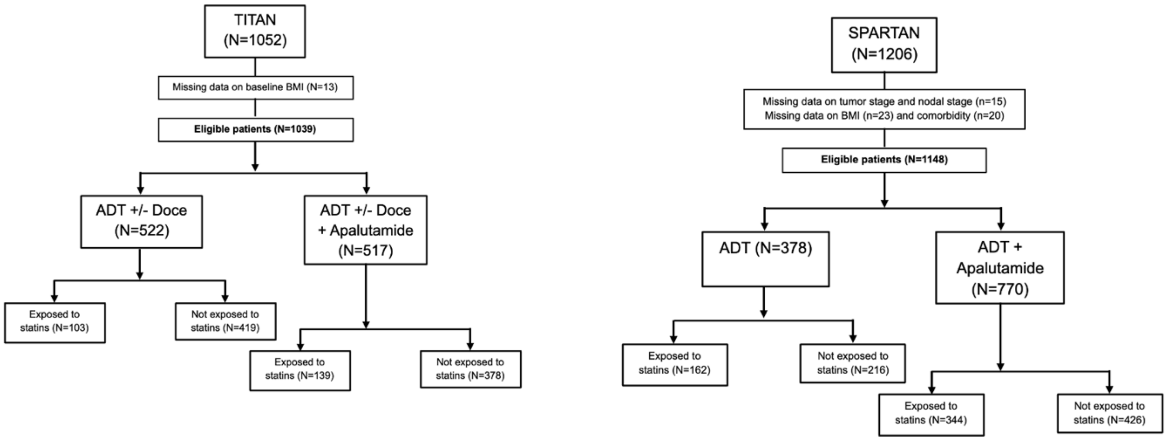

© 2025 Roy S et al. *JAMA Network Open*.

eTable 1: Multivariable Cox proportional hazard regression model summarizing association of statin exposure with OS among patients treated with and without apalutamide in the TITAN and SPARTAN trials in landmark populations at various timepoints, respectively.

Treatment group	Hazard ratio for statin exposure with 95% CI for 6-month landmark population	
	TITAN	SPARTAN
**Apalutamide**	0.51 (0.31-0.85)	0.59 (0.44-0.80)
**Placebo**	0.59 (0.36-0.97)	1.13 (0.75-1.71)
	**Hazard ratio for statin exposure with 95% CI for 12-month landmark population**	
**Apalutamide**	0.46 (0.26-0.79)	0.65 (0.48-0.88)
**Placebo**	0.62 (0.37-1.05)	1.16 (0.76-1.77)
	**Hazard ratio for statin exposure with 95% CI for 24-month landmark population**	
**Apalutamide**	0.48 (0.24-0.96)	0.65 (0.46-0.91)
**Placebo**	0.48 (0.23-1.00)	1.08 (0.67-1.72)

© 2025 Roy S et al. *JAMA Network Open*.

eTable 2: Distribution of organ systemic specific grade ≥3 adverse events in apalutamide treated patients stratified by exposure to statins.

Body System or Organ Class	Not Exposed to Statins		Exposed to Statins		Overall (N=1291)
	SPARTAN (N=428)	TITAN (N=378)	SPARTAN (N=346)	TITAN (N=139)	
Blood and lymphatic system disorders	7	9	4	3	23
Cardiac disorders	9	8	20	9	46
Eye disorders	3	1	8	1	13
Gastrointestinal disorders	14	7	12	2	35
General disorders and administration site conditions	8	11	5	2	26
Hepatobiliary disorders	1	0	1	2	5
Immune system disorders	1	0	1	0	2
Infections and infestations	17	9	26	4	56
Injury, poisoning and procedural complications	13	4	11	5	33
Investigations	12	10	10	2	34
Metabolism and nutrition disorders	10	7	17	5	49
Musculoskeletal and connective tissue disorders	9	25	12	3	49
Neoplasms benign, malignant and unspecified (incl cysts and polyps)	16	7	8	2	33
Nervous system disorders	11	13	17	7	48
Renal and urinary disorders	27	10	19	3	59
Respiratory, thoracic and mediastinal disorders	10	4	7	5	26
Skin and subcutaneous tissue disorders	23	24	12	8	67
Vascular disorders	49	22	41	11	123
Ear and labyrinth disorders	0	0	0	1	1
Psychiatric disorders	0	3	0	0	3
Reproductive system and breast disorders	0	0	1	0	2
Product issues	0	0	1	0	1

© 2025 Roy S et al. *JAMA Network Open*.

eTable 3: Distribution of organ systemic specific grade ≥3 adverse events in placebo treated patients stratified by exposure to statins.

Body System or Organ Class	Not statin Exposed		Statin Exposed		Overall (N=902)
	SPARTAN (N=218)	TITAN (N=419)	SPARTAN (N=162)	TITAN (N=103)	
Blood and lymphatic system disorders	3	10	1	2	16
Cardiac disorders	3	5	8	4	20
Ear and labyrinth disorders	1	0	0	0	1
Eye disorders	3	1	3	2	9
Gastrointestinal disorders	4	1	9	3	17
General disorders and administration site conditions	2	14	3	2	21
Hepatobiliary disorders	2	1	1	0	4
Immune system disorders	1	0	0	0	1
Infections and infestations	5	20	6	5	36
Injury, poisoning and procedural complications	4	7	1	4	16
Metabolism and nutrition disorders	3	15	3	5	26
Musculoskeletal and connective tissue disorders	9	31	4	8	52
Neoplasms benign, malignant and unspecified (incl cysts and polyps)	6	7	1	1	19
Nervous system disorders	4	17	3	4	28
Renal and urinary disorders	21	9	12	1	43
Respiratory, thoracic and mediastinal disorders	2	7	4	2	15
Skin and subcutaneous tissue disorders	1	8	0	3	12
Vascular disorders	27	17	17	8	69
Investigations	0	22	1	1	25
Psychiatric disorders	0	3	0	0	3
Reproductive system and breast disorders	1	1	1	0	2

© 2025 Roy S et al. *JAMA Network Open*.

eTable 4: Overall and trial specific association of statin exposure with risk of grade ≥3 cardiac adverse events (SAE) across two different treatment regimens using logistic regression model.

Treatment group	Odds ratio for statin exposure with 95% confidence intervals		
	**Overall**	**TITAN**	**SPARTAN**
Apalutamide	2.85 (1.50-5.58)	3.19 (1.09-9.42)	2.72 (1.20-6.54)
Placebo	2.46 (0.93-6.79)	2.19 (0.49-9.20)	2.75 (0.72-13.37)

© 2025 Roy S et al. *JAMA Network Open*.
