# Supplementary material for: Statin Use in Patients With Advanced Prostate Cancer in the TITAN and SPARTAN Trials
Source: JAMA Netw Open. 2025 Aug 20;8(8):e2527988. doi: 10.1001/jamanetworkopen.2025.27988 (PMC12368675; doi:10.1001/jamanetworkopen.2025.27988)
Supplement: Supplement 2. — Data Sharing Statement [file jamanetwopen-e2527988-s002.pdf]

Data Sharing Statement

Roy. Statin Use in Patients With Advanced Prostate Cancer in the TITAN and SPARTAN Trials. *JAMA Netw Open*. Published August 20, 2025.
doi:10.1001/jamanetworkopen.2025.27988

Data
Data available: This study was carried out under Yale Open Data Access (YODA) Project # 2023-5173 and used data obtained from the Yale University Open Data Access Project, which has an agreement with Janssen Research & Development, L.L.C, and the primary sponsor of the SPARTAN trial. Thus, the data would not be shared by the authors. The interpretation and reporting of research using this data are solely the responsibility of the authors and do not necessarily represent the official views of the Yale University Open Data Access Project or JANSSEN RESEARCH & DEVELOPMENT, L.L.C.
